# A nomogram to predict lateral lymph node metastases in lateral neck in patients with medullary thyroid cancer

**DOI:** 10.3389/fendo.2022.902546

**Published:** 2022-08-16

**Authors:** Lichao Jin, Xiwei Zhang, Song Ni, Dangui Yan, Minjie Wang, Zhengjiang Li, Shaoyan Liu, Changming An

**Affiliations:** ^1^ Department of Head and Neck Surgery, National Cancer Center/National Clinical Research Center for Cancer/Cancer Hospital, Chinese Academy of Medical Sciences and Peking Union Medical College, Beijing, China; ^2^ Department of Clinical Laboratory, National Cancer Center/National Clinical Research Center for Cancer/Cancer Hospital, Chinese Academy of Medical Sciences and Peking Union Medical College, Beijing, China

**Keywords:** medullary thyroid cancer, lateral lymph node metastases, nomogram, prophylactic lateral neck dissection, calcitonin,

## Abstract

**Background:**

Medullary thyroid cancer (MTC) can only be cured by surgery, but the management of lateral lymph nodes is controversial, especially for patients with cN0+cN1a. To address this challenge, we developed a multivariate logistic regression model to predict lateral lymph node metastases (LNM).

**Methods:**

We retrospectively collected clinical data from 124 consecutive MTC patients who underwent initial surgery at our institution. The data of 82 patients (from 2010 to 2018) and 42 patients (from January 2019 to November 2019) were used as the training set for building the model and as the test set for validating the model, respectively.

**Results:**

In the training group, the multivariate analyses indicated that male and MTC patients with higher preoperative basal calcitonin levels were more likely to have lateral LNM (P = 0.007 and 0.005, respectively). Multifocal lesions and suspected lateral LNM in preoperative ultrasound (US) were independent risk factors (P = 0.032 and 0.002, respectively). The identified risk factors were incorporated into a multivariate logistic regression model to generate the nomogram, which showed good discrimination (C-index = 0.963, 95% confidence interval [CI]: 0.9286–0.9972). Our model was validated with an excellent result in the test set and even superior to the training set (C-index = 0.964, 95% CI: 0.9121–1.000).

**Conclusion:**

Higher preoperative basal calcitonin level, male sex, multifocal lesions, and lateral lymph node involvement suspicion on US are risk factors for lateral LNM. Our model and nomogram will objectively and accurately predict lateral LNM in patients with MTC.

## Introduction

Medullary thyroid cancer (MTC) is a relatively rare malignant tumor that accounts for 1%–2% of all thyroid cancers (1). It is a life-threatening disease with a prognosis between differentiated thyroid cancer and anaplastic thyroid cancer in terms of life expectancy (2, 3). In addition, MTC originates from parafollicular epithelial cells and is capable of specifically secreting calcitonin (4, 5), which is a highly specific tumor marker with preoperative and postoperative responses that can reflect the tumor load and treatment outcomes (2, 3, 6, 7).

Although targeted therapy is currently approved for advanced MTC, surgery is still the first choice and only proven method for curing MTC (1, 4). The outcome of the initial surgical treatment is a key factor in the prognosis. Patients who achieve biochemical cure after surgery have a 10-year survival rate of 95% to 97% (8), while patients with persistently elevated calcitonin(Ctn) have a 5- and 10-year survival rate of 80% to 86% and 70% (9), respectively. However, the extent of the initial surgical treatment remains controversial. According to the American Association of Endocrine Surgeons (AAES) and the American Thyroid Association (ATA) guidelines, treatment opinions on primary tumors tend to be consistent, that is, total thyroidectomy and prophylactic central neck dissection is recommended as the initial surgical treatment (5, 10). Moreover, therapeutic lateral lymph node dissection should be performed when a fine needle biopsy (FNB) is positive.

The controversy mainly focuses on whether patients without obvious evidence supporting lateral lymph node metastases (LNM) need to undergo prophylactic lateral neck dissection (11). The ATA guidelines 2015 affirmed the role of preoperative serum calcitonin on whether patients with cN0 + cN1a should undergo lateral neck dissection, but no clear cut-off value was provided (5). Machens et al. showed that when preoperative serum calcitonin exceeded 200 pg/ml, bilateral neck dissection could reduce the number of reoperations (12). However, a nationwide population-based study in Norway showed that extrathyroidal extension and no biochemical cure instead of serum basal calcitonin alone might predict the need for prophylactic lateral lymph node dissection in patients with MTC (13).

Aim of the present study is to establish a multivariate logistic regression model based on neck ultrasound examination, serum Ctn levels and other prognostic factors useful to predict the risk of lateral LNM and identify those patients likely to benefit for lateral neck dissection. This model was further presented as a nomogram and provided specific recommendations on whether patients with MTC should undergo lateral neck dissection.

## Materials and method

### Patients and study design

A retrospective study was conducted on a primary cohort of patients who were pathologically diagnosed with MTC at the Cancer Hospital Chinese Academy of Medical Sciences (Beijing, China) between 2010 and 2018. The inclusion criteria were as follows: 1) no history of previous anticancer therapy, 2) preoperative and postoperative serum calcitonin measurements were available for analysis, and 3) preoperative thyroid ultrasound and pathology reports were obtained and reviewed. Patients with a history of thyroid surgery were excluded from the study. Individual patients that were treated with total thyroidectomy and bilateral central neck dissection without achieving biochemical cure were not included in the study due to possible persistent disease in lateral lymph nodes. The aim of this study was to construct a preoperative non-invasive assessment protocol, and therefore FNB results were not included as a study factor. From January 2019 to December 2019, an independent cohort of consecutive patients was included in the validation cohort using the same inclusion and exclusion criteria.

This study was approved by the institutional ethics committee of the Cancer Hospital, Chinese Academy of Medical Sciences (NCC-006628). Informed patients’ consent was obtained before surgery.

### Diagnosis and treatment

According to Chinese clinical guidelines for thyroid cancer, patients diagnosed with MTC underwent thyroidectomy and central neck dissection, while those with a preoperative diagnosis of cN1b underwent lateral neck dissection. Decision for lateral neck dissection was based on the patient’s serum calcitonin, preoperative ultrasound, computed tomography, FNB and *RET* mutations. Lateral neck dissection included the removal of lymph nodes and surrounding tissues (levels II–IV or II–V). All patients received only thyroid hormone replacement therapy after surgery, without targeted therapy or immunotherapy. If recurrence or distant metastasis occurred during follow-up, the corresponding treatment was given.

Based on pathological results, tumor extent was classified into two groups according to the presence of LNM: 1) no LNM or central LNM, and 2) lateral LNM (including ipsilateral LNM and contralateral LNM). Disease staging was classified according to the 8th edition of the American Joint Cancer Committee (AJCC) system.

### Ultrasound and measurement of serum calcitonin

The preoperative ultrasound was performed by the sonographer in our hospital, and the results were recorded according to the official report issued. From the ultrasound reports, we obtained information on the primary tumor location, diameter (in cm), total number of foci, bilateral lesions, central compartment nodal metastases, and lateral compartment nodal metastases.

The serum calcitonin level was measured with a two-site immunoradiometric assay using a commercial kit (MEDGENIX CT-U.S.-IRMA kit, BioSource Europe S.A., Belgium). The minimum detectable limit was 0.05 pg/mL. Serum calcitonin levels were checked on the first day and 1 month, 3 months, and 6 months after surgery and every 6 months thereafter. All enrolled patients were followed up for at least 6 months. Individual patients who were lost to follow-up at 6 months after surgery and whose calcitonin levels had decreased to normal and no suspicious LNM was seen on check up ultrasound were also included in the group as biochemically cured.

### Statistical analysis

Statistical analyses to identify risk factors were performed using SPSS (version 24.0; IBM Corp., Armonk, NY, USA). Categorical variables were grouped based on clinical findings, and decisions on the groups were made before modeling. The results were compared using the χ^2^ test or Fisher’s exact test. Continuous variables were compared using the *t*-test, or Mann–Whitney U test for variables with skewed distribution. Logistic regression analysis was performed for multivariate analyses using the forward and likelihood ratio (LR) methods.

A nomogram was formulated based on the results of multivariate analysis and using the rms package in R version 4.0.3 (http://www.r-project.org/). A final model selection was performed using a forward step-down selection process using the Akaike information criterion. The performance of the nomogram was measured using the concordance index (C-index) and assessed by comparing the nomogram-predicted versus observed lateral LNM probability. Bootstraps with 1,000 resamples were used for these analyses. A larger C-index indicates a more accurate prognostic prediction. During the external validation of the nomogram, the total points of each patient in the validation cohort were calculated according to the established nomogram, the logistic regression in this cohort was performed using the total points as a factor, and the C-index and calibration curve were derived based on the regression analysis. Statistical significance was set at p < 0.05. The related computerized programs for nomograms with R are listed in the Appendix.

## Results

### Characteristics of patients

From the beginning of 2010 to the end of 2018, a total of 87 patients were diagnosed with MTC and treated for the first time in our hospital. Five patients whose postoperative pathology showed no lateral LNM were excluded because they were lost to follow-up or did not achieve biochemical cure. The remaining 82 patients were included in the primary cohort. For the validation cohort, we studied 49 consecutive cases of MTC in 2019; seven cases did not meet the inclusion criteria, and remained 42 patients were eventually included in the validation cohort.

The clinical characteristics of patients are shown in **Table 1**. In the primary cohort, the pathologic lymph node status of 35 (42.7%) patients was pN0 and pN1a, indicating that no lateral cervical lymph node metastasis occurred, and 47 (57.3%) patients had at least one lateral LNM. Among the 42 patients with MTC in the validation cohort, 22 (52.4%) patients had lateral LNM. The mean age of the 82 patients with MTC in the primary cohort was 47.0 ± 13.0 years, of which 37 (45.1%) were male. The corresponding mean age in the validation cohort was 48.2 ± 14.5 years, of which 22 (47.2%) were male. The median primary tumor diameter was 1.8 (1.1–2.9) cm in the primary cohort and 1.8 (1.1–2.8) cm in the validation cohort. The median preoperative serum calcitonin concentration was 941.5 (236.0–2970.5) pg/mL and 924.6 (254.6–2820.3) pg/mL in the primary and validation cohorts, respectively. There was no significant difference in the baseline characteristics between two cohorts.

**Table 1:** Demographics and clinical characteristics of patients with pedullary thyroid cancer.

Demographic or Characteristic		Primary Cohort			Validation Cohort		
		N0+N1a (n=35)	N1b (n=47)	Total (n=82)	N0+N1a (n=20)	N1b (n=22)	Total (n=42)
Age (years, mean ± SD)		47.6 (13.6)	46.6 (12.6)	47.0 (13.0)	45.3 (12.5)	50.8 (16.0)	48.2 (14.5)
Sex	Male (%)	8 (22.9)	29 (61.7)	37 (45.1)	6 (30.0)	14 (63.6)	20 (47.6)
	Female (%)	27 (77.1)	18 (38.3)	45 (54.9)	14 (70.0)	8 (36.4)	22 (52.4)
Ultrasound	Maximum tumor diameter (cm, median, IQR)	1.3 (0.8-2.3)	2.2 (1.3-3.4)^#^	1.8 (1.1-2.9)	1.3 (1.0-2.3)	2.3 (1.6-3.6)	1.8 (1.1-2.8)
	Multiple foci (%)	3 (8.6)	16 (34.0)	19 (23.2)	4 (20.0)	6 (27.3)	10 (23.8)
	Bilateral lesions (%)	4 (11.4)	8 (17.0)	12 (14.6)	2 (10.0)	1 (4.5)	3 (7.1)
	Central LNM (%)	1 (2.9)	26 (55.3)	27 (32.9)	1 (5.0)	10 (45.5)	11 (26.2)
	Lateral LNM (%)	1 (2.9)	33 (70.2)	34 (41.5)	0 (0.0)	18 (81.8)	18 (42.9)
Preoperative basal serum calcitonin (pg/mL, median, IQR)		233.0 (77.0-622.0)	2000.0 (901.0-4880.0)	941.5 (236.0-2970.5)	279.6 (84.5-549.5)	1979.0 (936.3-4280.5)	924.6 (254.6.-2820.3)
Extent of neck dissection (%)	Central dissection	35 (100.0)	47 (100.0)	82 (100.0)	20 (100.0)	22 (100.0)	42 (100.0)
	Ipsilateral lateral dissection	10 (28.6)	44 (93.6)	54 (65.9)	1 (5.0)	22 (100.0)	23 (54.8)
	Contralateral lateral dissection	3 (8.6)	30 (63.8)	33 (40.2)	0 (0.0)	13 (59.1)	13 (31.0)
Number of LNMs (pathology, mean ± SD)	Central compartment	0.4 (1.1)	7.9 (8.2)	4.7 (7.2)	1.2 (2.2)	5.0 (3.2)	3.1 (3.3)
	Ipsilateral lateral compartment	/	11.4 (12.2)	6.5 (10.8)	/	12.5 (9.8)	6.6 (9.5)
	Contralateral lateral compartment		4.0 (10.3)	2.3 (8.0)		1.6 (3.4)	0.8 (2.6)
	Total	0.4 (1.1)	23.3 (26.0)	13.5 (22.6)	1.2 (2.2)	19.1 (11.7)	10.5 (12.4)

LN, lymph nodes; LNM, lymph node metastasis.

^#^A missing value.

### Independent prognostic factors in the primary cohort

The results of the univariate analysis are shown in **Table 2**. The independent risk factors related to lateral LNM after multivariate analysis included sex, primary tumor diameter under ultrasound, number of foci under ultrasound, ultrasound findings on lateral LNM and calcitonin levels.

**Table 2:** Univariate analysis and multivariate analysis of lateral LNM in the primary cohort.

Variable		Univariate analysis			Multivariate analysis^#^		
		OR	95%CI	*P*	OR	95%CI	*P*
Male		5.438	2.033-14.547	0.001	13.860	2.086-92.108	0.007
Age		0.994	0.960-1.208	0.721			0.628
Ln_Ctn		2.337	1.577-3.462	<0.001	2.659	1.342-5.271	0.005
Ultrasound	Maximum diameter of primary focus	1.068	1.021-1.118	0.004			0.088
	Multiple foci	5.505	1.458-20.782	0.012	12.033	1.232-117.537	0.032
	Bilateral lesions	1.590	0.438-5.773	0.481			0.162
	Central LNM	42.095	5.312-333.615	<0.001			0.108
	Lateral LNM	80.143	9.967-644.410	<0.001	316.344	8.494-11781.351	0.002

Ctn, Calcitonin; Ln, natural logarithm; LNM, lymph node metastasis.

^#^The logistic regression model used the Forward, LR method to calculate OR and 95% CI of all variables.

### Prognostic nomogram for lateral LNM

**Figure 1:**
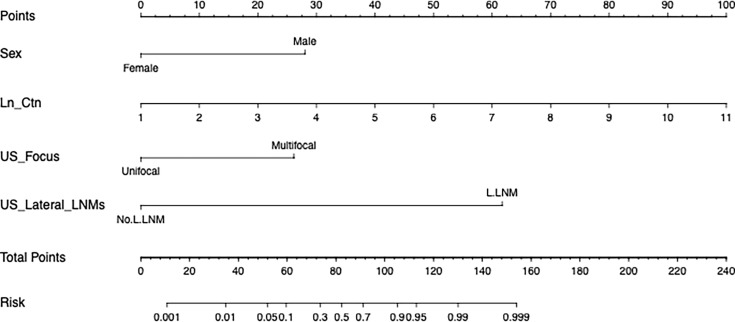
A nomogram for lateral cervical lymph node metastasis of medullary thyroid cancer. (To use this, locate a patient’s value on each variable axis and draw a line up to determine the number of points earned for each variable value. The sum of these numbers is on the total points axis, and then draw a line below the survival axis Line to determine the possibility of lateral cervical lymph node metastasis. Sex, the patient’s physiological gender; Ln_Ctn, the natural logarithm of the patient’s preoperative serum calcitonin; US_Focus, the number of suspicious nodules indicated by the patient’s preoperative ultrasound; US_Lateral_LNM, preoperative Ultrasound revealed suspicious metastasis of lateral cervical lymph nodes.).

**Figure 2:**
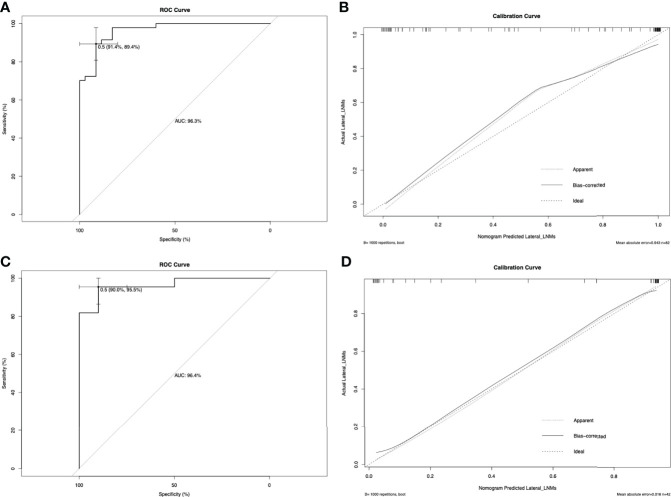
The ROC curve for predicting lateral neck lymph node metastasis in the primary cohort **(A)** and in the validation cohort **(C)**. In this study, metastasis of the lateral neck lymph node was used as the outcome variable, and metastasis was marked as 1, and non-metastasis was marked as 0. The ROC curve is drawn based on the predicted value of the nomogram and the actual situation of lateral cervical lymph node metastasis. The calibration curve for predicting patient lateral neck lymph node metastasis in the primary cohort **(B)** and in the validation cohort **(D)**. Nomogram-predicted probability of lateral neck LNM is plotted on the x-axis; actual lateral neck LNM is plotted on the y-axis.

**Figure 1** shows a prognostic nomogram that integrates all important lateral cervical lymph node independent factors. The C-index predicted by logistic regression was 0.963 (95% confidence interval [CI], 0.9286–0.9972) (**Figure 2A**). The calibration map 312 of lateral neck lymph node metastasis showed the best 313 concordance between the nomogram prediction and actual observations (**Figure 2C**).

### Comparison of predictive accuracy between the nomogram and a single independent factor

As shown in **Table 2**, the risk ratio of lateral LNM suggested by ultrasound was higher than that of other factors. We further compared the accuracy to predict LNM with nomogram and the ultrasound. The C-index of ultrasound results used to predict lateral LNM was 0.837, which was significantly lower than that of the nomogram (0.963; P < 0.001).

Serum calcitonin is recommended in the guidelines for predicting lateral cervical lymph node metastasis, and it is also the only continuous variable with statistical significance (**Table 2**). Therefore, we also compared the predictive ability of serum calcitonin levels and the nomogram. Because the logistic model requires a linear relationship between the continuous independent variable and the logit conversion value of the dependent variable, the value of serum calcitonin has undergone a natural logarithmic conversion, expressed as LN_Ctn. The C-index of LN_Ctn was 0.851, which was significantly lower than that of the nomogram (P < 0.001).

### Predictive accuracy of the nomogram in the validation cohort

In the validation cohort, the C-index of the nomogram used to predict lateral LNM was 0.964 (95% CI, 0.9121–1.000), and the calibration curve showed good concordance between the predicted and observed values of the lateral cervical lymph node (**Figures 2B, D**). Similarly, the C-indexes of preoperative serum calcitonin and ultrasound results to predict lateral cervical lymph node metastasis were 0.886 and 0.909, respectively. Compared with the prediction results of the nomogram, there was a significant difference (P < 0.001).

## Discussion

In this study, we analyzed the correlation between preoperative serum calcitonin levels, ultrasound results, and baseline characteristics of MTC patients with lateral LNM. Through univariate and multivariate analyses of the above variables, we found that preoperative serum calcitonin level was an independent risk factor for lateral LNM in patients with MTC. In the preoperative ultrasound results, multiple foci and ultrasound findings suggesting lateral LNM indicated that the postoperative pathology was more likely to be N1b. Finally, based on these findings, we established a multivariate logistic regression model to evaluate the possibility of lateral LNM in MTC patients before surgery and further visualized our model in a nomogram for clinical practice. The prediction of the nomogram is superior to traditional prediction based on ultrasonography or preoperative serum calcitonin levels. More importantly, we tested our model with an independent MTC cohort and obtained similar results, which further proved the reliability and applicability of our model.

The prevailing opinion is difficult to agree on the issue of whether patients with MTC without clear evidence of lateral LNM in the preoperative evaluation should undergo lateral neck dissection. This is partly because the initial surgical treatment is closely related to the prognosis (2, 14). Achieving biochemical cure is the main goal of surgical treatment for MTC patients. The 10-year survival rate of biochemically cured MTC patients could be 25% or more higher than patients with persistent hypercalcitoninemia (8). Avoiding secondary surgery can reduce the financial burden and psychological stress for patients. Lateral neck dissection, on the other hand, implies greater surgical extent and surgical trauma. Since the incision is located in the neck, poor scarring can have aesthetic impact on the quality of life. At the same time, risk of complications is higher when more extensive surgery is performed, such as the permanent recurrent laryngeal nerve palsy, permanent hypocalcemia, hematoma, shoulder syndrome, Horner syndrome, and chyle leakage (15).

Our study showed that tested nomogram had good predictive ability for detection of LNM. In the primary cohort, the negative and positive predictive values were 0.865 and 0.911, respectively. These results were verified in the validation cohort, with the negative and positive predictive values at 0.900 and 0.955, respectively. Our model might help optimize the extent of surgery regarding lateral neck dissections. The negative likelihood ratio (LR) is the ratio of the false-negative and true-negative rates detected in clinical tests. The literature data suggest that a negative LR of a negative test less than 0.2 is good at ruling out a diagnosis and less than 0.1 is excellent (16–18). The negative LR was 0.12 and 0.09 in the primary and validation cohorts, respectively. Our model can effectively predict the possibility of lateral LNM in MTC patients with cN0 + cN1a, which means it could be useful to avoid unnecessary lateral neck dissection. On the other hand, it could be a helpful tool to identify those patients without a clear pre-surgical evidence of lateral LNM likely to benefit from a more extensive surgery.

Our research confirms that there is a positive correlation between preoperative serum calcitonin levels and the risk of lateral cervical lymph node metastasis. The greater the serum calcitonin level, the greater the possibility of lateral cervical lymph node metastasis. The odds ratio was 2.337 (1.577–3.462) in the univariate analysis and 2.659 (1.342–5.271) in the multivariate analysis, which are consistent with the results reported in the literature (19). Based on the study of the relationship between preoperative serum calcitonin levels and pathology findings, Machens et al. emphasized that preoperative serum calcitonin levels of more than 20, 50, and 200 pg/mL are associated with ipsilateral central and lateral LNM, contralateral central LNM, and contralateral lateral LNM, respectively (12). However, previous studies have confirmed that preoperative serum calcitonin levels are related to many factors, such as the diameter of the primary tumor and the malignancy of the tumor (19–21). Even when serum calcitonin levels are hundreds of times higher than the upper limit of normal, patients may not benefit from lateral neck dissection. In the primary cohort, 20 of 35 patients with pN0 had preoperative serum calcitonin levels >200 pg/mL. Two of them had calcitonin levels exceeding 8,000 pg/mL before the operation, but no lateral LNM were found in pathology. According to our model prediction, 18 of these 20 patients had a predictive value of less than 0.5, which means prophylactic lateral neck dissection were not required. The model could effectively avoid 90% of unnecessary lateral neck dissections. In the validation cohort, there were 20 patients with pN0, 11 of whom had a calcitonin level of >200 pg/mL. Only two of these 11 patients have a model prediction value of more than 0.5, which could have reduced unnecessary lateral neck dissection by 81.8%.

Preoperative ultrasound evaluation is the most important preoperative imaging study (5). However, the accuracy of ultrasound is highly dependent on experience and technical limitations (22, 23). False-negative results produced by preoperative ultrasound may lead to improper surgical extent, thereby increasing the risk of recurrence and poor prognosis. Based on previous ultrasound research results, the risk factors in our initial model included the size of the primary tumor, multifocality, bilateral pattern, central lymph node metastasis, and lateral LNM (24–26). After analysis, only multifocality and lateral LNM were statistically significant. Possibly, because the diameter of the primary lesion is closely related to the preoperative serum calcitonin level. After the inclusion of calcitonin, the primary lesion was eliminated as a related variable.

To use this nomogram, the value of the MTC patient is on the corresponding variable axis, and a vertical line is drawn up to obtain the total number of points. Then, all the points of the variable are added, and the sum of these numbers is assigned in the “risk” axis. The model recommends that when the risk is ≥0.5, patients with MTC should undergo lateral neck dissection. The model is not only applicable to patients without clear preoperative evidence to support lateral neck LNM, but also to patients with evidence. For example, the risk value assessed by the model will be greater than 0.5 in patients with ultrasound suspicion of lateral LNM, as long as the serum calcitonin level exceeds 20 pg/ml. And we know that the vast majority of MTC patients who progress to lateral LNM generally have serum calcitonin that is above or even well above this standard.

This study had several limitations. First, this was a retrospective study, and the nomogram was established based on data obtained from a single center. Second, this study did not distinguish between MTC types. Whether the model is applicable to hereditary MTC requires further verification. Third, according to previous studies, the 10-year survival rate of patients with biochemically cured MTC is 90%.

We developed a nomogram based on the identified risk factors, because it can make the data easier to visualize and use in clinical settings. This model can facilitate communication between surgeons and their patients or patients’ families in formulating surgical plans and prognostic analysis.

## Data availability statement

The raw data supporting the conclusions of this article will be made available by the authors, without undue reservation.

## Ethics statement

The studies involving human participants were reviewed and approved by the institutional ethics committee of the Cancer Hospital, Chinese Academy of Medical Sciences. The patients/participants provided their written informed consent to participate in this study. Written informed consent was obtained from the individual(s) for the publication of any potentially identifiable images or data included in this article.

## Author contributions

LJ and XZ had full access to all of the data in the study and take responsibility for the integrity of the data and the accuracy of the data analysis. Concept and design: All authors. Acquisition, analysis, or interpretation of data: All authors. Drafting of the manuscript: All authors. Critical revision of the manuscript for important intellectual content: All authors. Statistical analysis: LJ, XZ. All authors contributed to the article and approved the submitted version.

## Funding

This study was supported by a research funding (LC2018A18) from the Cancer Foundation of China.

## Acknowledgments

The authors would like to acknowledge the colleagues in the Department of Head and Neck Surgery for their support with the data.

## Conflict of interest

The authors declare that the research was conducted in the absence of any commercial or financial relationships that could be construed as a potential conflict of interest.

## Publisher’s note

All claims expressed in this article are solely those of the authors and do not necessarily represent those of their affiliated organizations, or those of the publisher, the editors and the reviewers. Any product that may be evaluated in this article, or claim that may be made by its manufacturer, is not guaranteed or endorsed by the publisher.
